# Diagnostic exome-based preconception carrier testing in consanguineous couples: results from the first 100 couples in clinical practice

**DOI:** 10.1038/s41436-021-01116-x

**Published:** 2021-03-19

**Authors:** Suzanne C. E. H. Sallevelt, Alexander P. A. Stegmann, Bart de Koning, Crool Velter, Anja Steyls, Melanie van Esch, Phillis Lakeman, Helger Yntema, Masoud Zamani Esteki, Christine E. M. de Die-Smulders, Christian Gilissen, Arthur van den Wijngaard, Han G. Brunner, Aimée D. C. Paulussen

**Affiliations:** 1 Department of Clinical Genetics, Maastricht University Medical Center+ (MUMC+), Maastricht, The Netherlands; 2 Research School for Developmental Biology (GROW), Maastricht University, Maastricht, The Netherlands; 3 Amsterdam UMC, Department of Clinical Genetics, Amsterdam Reproduction and Development Research Institute, University of Amsterdam, Amsterdam, The Netherlands; 4 Department of Human Genetics, Radboud University Nijmegen Medical Centre, Nijmegen, The Netherlands; 5 Donders center for Neuroscience, Radboudumc, Nijmegen, The Netherlands; 6 MHENS school of Neuroscience, Maastricht University, Maastricht, The Netherlands

## Abstract

**Purpose:**

Consanguineous couples are at increased risk of being heterozygous for the same autosomal recessive (AR) disorder(s), with a 25% risk of affected offspring as a consequence. Until recently, comprehensive preconception carrier testing (PCT) for AR disorders was unavailable in routine diagnostics. Here we developed and implemented such a test in routine clinical care.

**Methods:**

We performed exome sequencing (ES) for 100 consanguineous couples. For each couple, rare variants that could give rise to biallelic variants in offspring were selected. These variants were subsequently filtered against a gene panel consisting of ~2,000 genes associated with known AR disorders (OMIM-based). Remaining variants were classified according to American College of Medical Genetics and Genomics/Association for Molecular Pathology (ACMG/AMP) guidelines, after which only likely pathogenic and pathogenic (class IV/V) variants, present in both partners, were reported.

**Results:**

In 28 of 100 tested consanguineous couples (28%), likely pathogenic and pathogenic variants not previously known in the couple or their family were reported conferring 25% risk of affected offspring.

**Conclusion:**

ES-based PCT provides a powerful diagnostic tool to identify AR disease carrier status in consanguineous couples. Outcomes provided significant reproductive choices for a higher proportion of these couples than previous tests.

## INTRODUCTION

Autosomal recessive (AR) disease, caused by biallelic pathogenic variants, is generally associated with severe phenotypes and although individually rare, collectively contributes significantly to morbidity and mortality, often in infants and children.^1^

Each individual is estimated to be heterozygous for up to seven AR pathogenic variants associated with severe disease.^1^ When both partners of a couple carry a pathogenic variant in the same gene, they have a 25% risk of having affected offspring.^2^ The risk in nonrelated outbred partners without a family history of disease depends mainly on variant population frequencies related to their ethnic and/or geographical origins. Consanguineous partners have an additional risk, as they share more genetic material than nonrelated partners, which correlates with the inbreeding coefficient *Ϝ*. For a first cousin relationship, *Ϝ* is 0.0625, corresponding with 12.5% regions of homozygosity (RoH) across the genome in offspring.^3^ Consanguinity thus is a reproductive risk for transferral of AR disease.^3^ In genetic counseling, empiric risk estimates of 2–2.5% additional risk of a congenital disorder in offspring are used for first-degree cousin couples compared with nonconsanguineous couples with a baseline risk of ~2.5% in Europe^4,5^ (eurocat). However, studies assessing these risks are mostly small, of varying design, and/or based on diagnoses in affected offspring (e.g., neonates with major congenital anomalies).^6^ To the best of our knowledge systematic studies are scarce, partly due to the lack of a comprehensive carrier test. A recent study using exome sequencing (ES) data to estimate the impact of consanguinity on the incidence of intellectual disability suggests that the additional genetic risk associated with consanguinity may be higher than previously thought.^5^

The percentage of consanguineous marriages in specific parts of the world, such as the Middle East, West and South Asia, Northern Africa, and parts of Southern Europe ranges from 20% to 50%.^7^ It reflects traditions in many communities worldwide offering social and economic advantages.^8–10^ Although such marital practices are less common in Western European societies, increasing migration has led to increased distribution of consanguinity and its recognition as a potential factor in disease incidence and risk assessment.^11,12^ Preconception risk assessment enables consanguineous couples to make informed reproductive decisions, including options to avoid disease transmission such as prenatal diagnosis (PND) or preimplantation genetic testing (PGT). Relevant for clinical practice is the fact that consanguineous couples who are actually at 25% risk of having affected offspring, but without a positive family history, thus far could not be distinguished from consanguineous couples not at risk, except for relatively frequent disorders. Existing preconception carrier screening (PCS) panels generally contain limited numbers of genes and thus are less effective for the detection of the often (extremely) rare AR disease consanguineous couples may be at risk of^13^ (personal communication with centers offering smaller panels). Routine diagnostic ES has proven to be a very effective technology to identify new or rare disease genes—among these, many AR genes in consanguineous families.^14,15^ In a previous study, we presented pilot data and proof of principle of an unbiased ES-based preconception carrier test (PCT) in a research setting,^13^ showing its feasibility for application in diagnostics. This test was further developed toward a diagnostic, more automated pipeline and implemented in our clinical practice. Here we present the results of diagnostic PCT in 100 consanguineous couples.

## MATERIALS AND METHODS

### Subjects

Consanguineous couples were included from January 2018 until December 2019. All degrees of relatedness were accepted. Pregnant couples were accepted on case-by-case basis. Early pregnancy, enabling potential reproductive options following the PCT result, was a requirement. Couples were referred by clinical geneticists. The couples’ obstetrical histories ranged from none at all to previous nonaffected or affected or deceased offspring with or without a genetic diagnosis (Suppl. Table S1). Couples were extensively counseled, including about varying severity of the disorders in the test and the fact that PND/PGT may neither be available nor desired for every disease. All couples signed informed consent.

### Exome sequencing and PCT gene panel analysis

Routine diagnostic ES and variant calling were performed as described previously.^16^ ES data were filtered against genes reported in OMIM to be associated with AR disease (1,924–2,198 genes, depending on the gene panel version used at the time of inclusion (Suppl. Table S2 and PCT panel list; link includes previous versions). The panel was updated twice yearly by an expert panel of clinical geneticists and laboratory specialists. No stringent severity criteria were applied. Only genes with (in AR context) unclear or very mild phenotypes (e.g., woolly hair, OMIM 616760, *KRT25*) were excluded (see Suppl. Table S2 and Discussion for further elaboration). Variants with a dbSNP frequency >5% and homozygous variants in either individual of the couple were removed, after which both data sets were merged to select for genes in which both individuals share an identical or a nonidentical variant. As such, no information was available on individual carrier status results if the partner was not carrying a variant in the same gene. Consequently, the risk of detecting autosomal dominant (AD) disorders (associated with genes that can also cause AR disease) in the couple is very low. Variants were then classified by at least two laboratory specialists according to American College of Medical Genetics and Genomics/Association for Molecular Pathology (ACMG/AMP) guidelines, also including classified variants from our in-house database.^16–18^ Only variant combinations of class IV (likely pathogenic) and/or V (pathogenic) in the same gene were included in the final diagnostic couple report; variants of unknown significance were not reported. This means, for example, that previously unreported missense variants were not reported. For variants that were borderline based on the ACMG/AMP definitions for class IV/V variants, e.g., if only one reported case had been described, available evidence was assessed by the laboratory specialists for robustness (e.g., functional studies) and experts in the particular field were consulted if deemed appropriate. As the carrier frequency of spinal muscular atrophy (SMA) is generally high in all populations and in the majority of cases caused by the exon 7–8 deletion in the *SMN1* gene, a multiplex ligation-dependent probe amplification (MLPA) test was added using the MLPA P460 probe mix (©MRC Holland, Amsterdam, The Netherlands) to determine SMA carrier status.

Standard turnaround time was 100 days and expedited in pregnant cases.

## RESULTS

PCT was performed in 100 consanguineous couples (Suppl. Table S1). The majority of couples (56) were first cousins and 6 couples were even more closely related, e.g., double first cousins (Suppl. Table S1/Suppl. Fig. S1). The degree of consanguinity in the remainder varied from second and third-degree cousins to more distant relatedness, and/or couples in whom consanguinity was suspected partly based on the presence of one or more RoH in affected offspring. Twenty-nine percent of couples were of Turkish origin, 18% of indigenous Dutch, 14% of Afghan, 12% of Moroccan, and 10% of Syrian origin. Nineteen couples were enrolled in a PGT procedure because of an earlier genetic diagnosis. Another 23 couples had a known AR genetic diagnosis in one or more previous offspring. One further couple was a known carrier couple of an AR disorder without affected offspring. Fourteen couples presented with one or more affected children without a genetic diagnosis. Forty-three couples presented without a known history of AR disease/carrier state and without offspring affected by an unknown disease. These include couples with children affected by a known nonrecessive genetic diagnosis (i.e., chromosomal). For 5 of these 43 couples, their history included one or more unexplained intrauterine death (IUD) and/or (multiple) miscarriages. Five couples were pregnant at inclusion, with gestational ages between 7 and 10 weeks.

### Overall PCT diagnostic yield

**Fig. 1:**
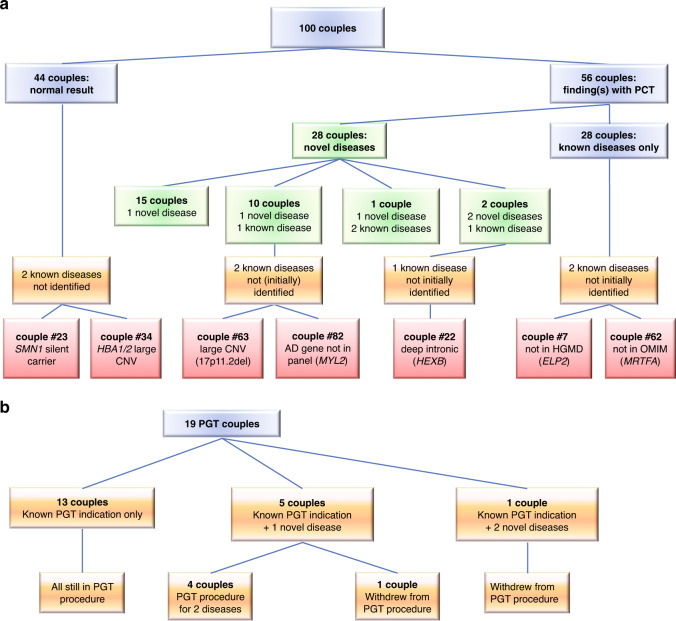
Preconception carrier test (PCT) results. (**a**) In the cohort of 100 consanguineous couples. Green boxes: novel detected variants, red boxes: variants not initially detected with the current PCT test design. (**b**) In the subgroup of couples undergoing preimplantation genetic testing (PGT). AD autosomal dominant, CNV copy-number variant, HGMD Human Gene Mutation Database.

PCT identified 30 novel (i.e., not previously known in the couple or their offspring) carrier couple states in 28/100 couples, resulting in a diagnostic yield for novel findings of 28% (Fig. 1a). The disease categories of the carrier states identified are listed in Suppl. Table S3, the most frequent being metabolic (*n* = 6), neurologic (*n* = 5), skeletal (*n* = 3), congenital deafness (*n* = 3), hepatic (*n* = 2), and ocular (*n* = 2).

In 13 cases, one (*n* = 11) or two (*n* = 2) novel additional carrier couple states were found in couples already known to be carrier couple of one, or two (couple 53), previously identified AR disease(s) (Fig. 1a).

In 6 of the 19 (32%) couples who were already enrolled in a PGT procedure, one (*n* = 5) or two (*n* = 1) additional carrier couple states were indeed identified by PCT (Fig. 1b).

Four novel findings in retrospect provided a likely explanation for the clinical phenotype of undiagnosed affected previous offspring (couples 9, 43, 56, and 100, Table 1). The previous child of couple 9 died at age 10 months, with epilepsy and developmental delay. DNA of the child was not preserved. PCT showed a *SUOX* variant in both parents, causative for sulfite oxidase deficiency, a lethal metabolic disorder matching the deceased child’s phenotype. Couple 43 lost a child due to a skeletal dysplasia. PCT showed a *TRIP11* variant in both parents, associated with achondrogenesis type 1a, matching the phenotype. The daughter of couple 56 died at 3 years of age, with a progressive disorder including epilepsy and deterioration of hearing and vision. PCT identified the couple as carriers of D-2-hydroxyglutaric aciduria (*D2HGD2*), a neurometabolic disorder matching the phenotype. In couple 100, whose daughter had died of hydrocephalus, PCT demonstrated a variant in the *CCDC88C* gene, causative for AR congenital hydrocephalus-1.

**Table 1:** Clinical and molecular data of the couples with PCT findings (confirmed and newly identified carrier couple states).

Couple number	Country of origin	Consanguinity	Family history	Number of shared pathogenic variants	Shared pathogenic variant (boldface: novel, detected by PCT)	Disease (for novel findings without previously affected child: most likely associated disease associated with identified variant, based on previous reports of identical or similar variant)
1	Turkey	Double 1st cousins	One healthy daughter	1	**Chr5(GRCh37):g.149359991C**>**T, NM_000112.3(SLC26A2):c.835C**>**T, p.(Arg279Trp)**	**Multiple epiphyseal dysplasia, type 4 (OMIM 226900)**
2	Turkey	Double 1st cousins	One daughter homozygous for *ATP8A2* variant	2	Chr13(GRCh37):g.26151250C>T, NM_016529.5(ATP8A2):c.1756C>T, p.(Arg586*) **Chr13(GRCh37):g.20763452A**>**G**, **NM_004004.5(GJB2):c.269T**>**C, p.(Leu90Pro)**	Cerebellar ataxia, mental retardation, and dysequilibrium syndrome 4 (OMIM 615268) **Autosomal recessive deafness type 1 A (OMIM 220290)**
4	Turkey	1st cousins	One daughter homozygous for *HGSNAT* variant, three healthy children	2	Chr8(GRCh37):g.43002207G>A, NG_009552.1(HGSNAT):c.234 + 1G>A, p.? **Chr1(GRCh37):g.94473807C**>**T, NM_000350.2(ABCA4):c.5882G**>**A, p.(Gly1961Glu)**	Mucopolysaccharidosis type IIIC, Sanfilippo C (OMIM 252930) **Stargardt disease 1 (OMIM 248200)**
6	Turkey	1st cousins	One son homozygous for *ACOX1* variant, died at age five years	2	Chr17(GRCh37):g.73956446G>A, NM_004035.6(ACOX1):c.280C>T, p.(Arg94*)	Peroxisomal acyl-CoA oxidase deficiency (OMIM 264470)
7	Turkey	2nd cousins	One daughter homozygous for *ELP2* variant, one healthy daughter	1	Chr18(GRCh37):g.33736538G>A, NM_001242875.2(ELP2):c.1580G>A, p.(Arg527Gln)	Mental retardation, autosomal recessive 58 (OMIM 616054)
9	Turkey	Distant	One daughter with epilepsy and intellectual disability, no diagnosis, died at age 10 months	1	**Chr12(GRCh37):g.56398257G**>**A, NM_000456.2(SUOX):c.1084G**>**A, p.(Gly362Ser)**	**Sulfite oxidase deficiency (OMIM 272300)**
10	Palestine	1st cousins	One deceased daughter homozygous for *SLC26A2* variant	1	Chr5(GRCh37):g.149360962_149360965del, NM_000112.3(SLC26A2):c.1806_1809del, p.(Thr603Serfs*5)	Achondrogenesis type 1B (OMIM 600972)
11	Netherlands	Distant	One son homozygous for *SGSH* variant	1	Chr17(GRCh37):g.78187614C>T, NM_000199.4(SGSH):c.734G>A, p.(Arg245His)	Mucopolysaccharidosis type IIIA (Sanfilippo type A) (OMIM 605270)
14	Pakistan	1st cousins	One daughter homozygous for *HBB* variant	1	Chr11(GRCh37):g.5247976_5247979dup, NM_000518.4(HBB):c.143_146dup, p.(Thr51Valfs*4)	β-thalassemia (OMIM 613985)
16	Netherlands	Distant	One son homozygous for *GAA* variant; multiple miscarriages; 10 weeks pregnant at inclusion	2	Chr17(GRCh37):g.78082327A>T, NM_000152.3(GAA):c.1115A>T, p.(His372Leu) Chr1(GRCh37):g.40756551dup, NM_005857.4(ZMPSTE24):c.1085dup, p.(Leu362Phefs*19)	Glycogen storage disease II (Pompe disease) (OMIM 232300) **Lethal restrictive dermopathy (OMIM 275210)**
21	Netherlands	Distant	One deceased son homozygous for *SFTPB* variant; one healthy daughter, one miscarriage	1	Chr2(GRCh37):g.85893772delinsTTC, NM_000542.3(SFTPB):c.397delinsGAA, p.(Pro133Glufs*95)	Pulmonary surfactant metabolism dysfunction 1 (OMIM 265120)
22	Turkey	1st cousins	One deceased son homozygous for *HEXB* variant, one healthy daughter	3	Chr2(GRCh37):g.1507755del, NM_000547.5(TPO):c.2422del, p.(Cys808Alafs*24) Chr15(GRCh37):g.83328380_83328383del, NM_001278512.1(AP3B2):c.3235_3238del, p.(Thr1079Serfs*7) Chr5(GRCh37):g.74016587_74016590dup, NG_009770.2(HEXB):c.1613 + 15_1613 + 18dup	**Thyroid dyshormonogenesis 2A (OMIM 274500)** **Early infantile epileptic encephalopathy 48 (OMIM 617276)** Sandhoff disease (OMIM 268800)
28	Morocco	Distant	One daughter homozygous for *OBSL1* variant	1	Chr2(GRCh37):g.220432786dup, NM_015311.2(OBSL1):c.1273dup, p.(Thr425Asnfs*40)	3M syndrome (OMIM 612921)
31	Afghanistan	1st cousins	Three healthy children, one IUD at 18 weeks	1	**Chr22(GRCh37):g.24896073A**>**G, NG_012858.2(UPB1):c.105-2A**>**G**	**β-ureidopropionase deficiency (OMIM 613161)**
32	Turkey	1st cousins	One son homozygous for *ACADVL* variant, one healthy son	1	Chr17(GRCh37):g.7127050G>A, NG_007975.1(ACADVL):c.1269 + 1G>A	VLCAD deficiency (OMIM 201475)
33	Syria	1st cousins	Two sons homozygous for *C12orf57* variant	1	Chr12(GRCh37):g.7053285A>G, NM_138425.3(C12orf57):c.1A>G	Temtamy syndrome (OMIM 218340)
36	Morocco	1st cousins	One son homozygous for *MBOAT7* variant, one healthy daughter	1	Chr12(GRCh37):g.7053285A>G, NM_024298.4(MBOAT7):c.458_459del, p.(Leu153Glnfs*142)	Mental retardation, autosomal recessive 57 (OMIM 617188)
37	Palestine	1st cousins	One son with congenital deafness	1	**Chr7(GRCh37):g.87041333C**>**T, NM_000443.3(ABCB4):c.2800G**>**A, p.(Ala934Thr)**	**Gallbladder disease 1 (LPAC) (OMIM 600803)**
38	Netherlands	2nd cousins	Son homozygous for *BLM* variant	1	Chr15(GRCh37):g.91328183C>T, NM_000057.3(BLM):c.2695C>T, p.(Arg899*)	Bloom syndrome (OMIM 210900)
43	Afghanistan	1st cousins	One deceased daughter with a skeletal dysplasia, diagnosis unknown	1	Chr14(GRCh37):g.92480711_92480714del, NM_004239.4(TRIP11):c.1031_1034del, p.(Arg344Lysfs*2)	**Achondrogenesis, type IA (OMIM 200600)**
44	Netherlands	Distant	One daughter with homozygous *MRPL44* variant	1	Chr2(GRCh37):g.224824538T>G, NM_022915.3(MRPL44):c.467T>G, p.(Leu156Arg)	Combined oxidative phosphorylation deficiency 16 (OMIM 615395)
45	Syria	1st cousins	One son homozygous for *SMN1* ex7–8 deletion	1	NM_000344.3(SMN1): ex7-8del	Spinal muscular atrophy 1 (OMIM 253300)
47	Morocco	2nd cousins	One daughter homozygous for *TGM1* variant	1	Chr14(GRCh37):g.24728366del, NM_000359.2(TGM1):c.1074del, p.(Ser358Argfs*26)	Ichthyosis, congenital, autosomal recessive 1 (OMIM 242300)
51	Turkey	Double 1st cousins	One daughter homozygous for *SCAPER* variant	2	Chr15(GRCh37):g.77057949_77057952del, NM_020843.3(SCAPER):c.1447_1450del, p.(Phe483Valfs*30) **Chr7(GRCh37):g.87041275G**>**T, NM_000443.3(ABCB4):c.2858C**>**A, p.(Ala953Asp)**	Intellectual developmental disorder and retinitis pigmentosa (OMIM 618195) **Cholestasis, progressive familial intrahepatic 3 (OMIM 602347)**
52	Turkey	1st cousins	One son, one daughter homozygous for *NEK9* variant, one healthy daughter	1	Chr14(GRCh37):g.75576537G>A, NM_033116.5(NEK9):c.1033C>T, p.(Arg345*)	Lethal congenital contracture syndrome 10 (OMIM 617022)
53	Yemen/Sudan	2nd cousins	One deceased daughter homozygous for *WDR73* and *IGHMBP2* variants	3	Chr11(GRCh37):g.68696686G>T, NM_002180.2(IGHMBP2):c.1096G>T, p.(Glu366*) Chr15(GRCh37):g.85186706del, NM_032856.3(WDR73):c.1132del, p.(Arg378Alafs*25) **Chr11(GRCh37):g.102991434C**>**T, NM_001080463.1(DYNC2H1):c.1151C**>**T, p.(Ala384Val)**	Charcot–Marie–Tooth disease, axonal, type 2S (OMIM 616155) Galloway–Mowat syndrome 1 (OMIM 251300) **Short-rib thoracic dysplasia 3 with or without polydactyly (OMIM 603297)**
54	Pakistan	3rd cousins	Two deceased sons homozygous for *VIPAS39* variant	1	Chr14(GRCh37):g.77910630del, NM_001193314.1(VIPAS39):c.559del, p.(Glu187Argfs*3)	Arthrogryposis, renal dysfunction, and cholestasis 2 (ARCS2) (OMIM 613404)
56	Syria	1st cousins	One deceased daughter, died at 3 years, progressive deterioration of hearing, seeing, epilepsy, no clinical diagnosis	1	Chr2(GRCh37):g.242683170del, NM_152783.4(D2HGDH):c.624del, p.(Gly209Glufs*31)	**D-2-hydroxyglutaric aciduria (OMIM 600721)**
61	Syria	1st cousins	Son homozygous for *HBB* variant	1	Chr11(GRCh37):g.5248232T>A, NM_000518.4(HBB):c.20A>T, p.(Glu7Val)	Sickle cell anemia (OMIM 603903)
62	Turkey	1st cousins	Son and daughter homozygous for *MRTFA* variant	1	Chr22(GRCh37):g.40815086dup, NM_020831.4(MRTFA):c.1356dup	Immunodeficiency 66 (OMIM 618847)
63	Syria	1st/2nd cousins	Son with Sjogren–Larsson syndrome (arr 17p11.2(19,447,016-19,655,447)x0, one healthy daughter	2^ϯ^	**Chr6(GRCh37):g.51609303A**>**G, NM_138694.3(PKHD1):c.10036T**>**C, p.(Cys3346Arg) maternal** **Chr6(GRCh37):g.51484077G**>**C, NM_138694.3(PKHD1):c.12027C**>G, p.(Tyr4009*) paternal both carry (arr 17p11.2(19,447,016-19,655,447)x1	**Polycystic kidney disease 4, with or without hepatic disease (OMIM 263200)** Sjogren–Larsson syndrome
65	Afghanistan	1st cousins	Two children homozygous for *TCIRG1* variant	1	Chr11(GRCh37):g.67811762dup, NM_006019.3(TCIRG1):c.971dup, p.(Cys324Trpfs*166)	Osteopetrosis, autosomal recessive 1 (OMIM 259700)
66	Iraq	1st cousins		2^ϯ^	**Chr16(GRCh37):g.3293447C**>**G, NM_000243.2(MEFV):c.2040G**>**C, p.(Met680Ile), paternal** **Chr16(GRCh37):g.3293407T**>**C, NM_000243.2(MEFV):c.2080A**>**G, p.(Met694Val), maternal**	**Familial Mediterranean fever, AR (OMIM 249100)**
70	Netherlands	3rd cousins	One child homozygous for *GJB2* variant	1	Chr13(GRCh37):g.20763686del, NM_004004.5(GJB2):c.35del, p.(Gly12Valfs*2)	Deafness, autosomal recessive 1A (OMIM 220290)
71	Turkey	1st cousins	One son homozygous for *PLA2G6* variant, one healthy daughter	2	Chr22(GRCh37):g.38536033dup, NM_003560.3(PLA2G6):c.753dup, p.(Asn252Glnfs*130) Chr1(GRCh37):g.152281672del, NM_002016.1(FLG):c.5690del, p.(His1897Profs*198)	Infantile neuroaxonal dystrophy 1 (OMIM 256600)/neurodegeneration with brain iron accumulation 2B (OMIM 610217) **Ichthyosis vulgaris (OMIM 146700)**
72	Netherlands	1st cousins		1	**Chr3(GRCh37):g.171431702G**>**A, NM_002662.4(PLD1):c.892C**>T, p.(Arg298*)	**Cardiac valvular defect, developmental (OMIM 212093)**
75	Unknown	1st cousins	Seven spontaneous abortions, one healthy son	1	**Chr12(GRCh37):g.110029107G**>**A, NM_000431.3(MVK):c.830G**>**A, p.(Arg277His)**	**Hyper-IgD syndrome (OMIM 260920)**
76	Netherlands	1st cousins	Deceased daughter homozygous for *CLPB* variant, one IUD at 16 weeks, one miscarriage; 7 weeks pregnant at inclusion	2	Chr11(GRCh37):g.72005169G>A, NM_030813.5(CLPB):c.1772C>T, p.(Ala591Val) **Chr16(GRCh37):g.84203896C**>**T, NM_178452.5(DNAAF1):c.1462C**>T, p.(Arg488*)	3-methylglutaconic aciduria, type VII, with cataracts, neurologic involvement and neutropenia (OMIM 616271) **Ciliary dyskinesia, primary, 13 (OMIM613193)**
77	Morocco	1st cousins	One healthy daughter	1	Chr11(GRCh37):g.93523799_93523800del, NM_004268.4(MED17):c.477_478del, p.(Leu160Ilefs*9)	**Microcephaly, postnatal progressive, with seizures and brain atrophy (OMIM 613668)**
78	Afghanistan	1st cousins	One termination of pregnancy homozygous for *WNT10B* and *PKP1* variants	2	Chr12(GRCh37):g.49360307del, NM_003394.3(WNT10B):c.741del, p.(Cys247*) Chr1(GRCh37):g.201288984C>T, NM_000299.3(PKP1):c.1273C>T, p.(Gln425*)	Split-hand/foot malformation 6 (OMIM 225300) Ectodermal dysplasia/skin fragility syndrome (OMIM 604536)
80	Morocco	Distant	One daughter with ID and epilepsy	1	Chr15(GRCh37):g.28171357_28171358del, NM_000275.2(OCA2):c.1994_1995del, p.(Ala665Glyfs*4)	**Albinism, oculocutaneous, type II (OMIM 203200)**
81	Morocco	1st cousins	One healthy daughter, one son homozygous for *SLC13A5* variant	1	Chr17(GRCh37):g.6597517C>T, NG_034220.1(SLC13A5):c.1056-1G>A, p.?	Epileptic encephalopathy, early infantile, 25 (OMIM 615905)
82	Netherlands	1st cousins	One deceased daughter homozygous for *MYL2* variant	2	Chr12(GRCh37):g.111348980C>G, NG_007554.1(MYL2):c.403-1G>C, p.? **Chr11(GRCh37):g.68707139T>G, NM_002180.2(IGHMBP2):c.2922T>G, p.(Asp974Glu)**	Cardiomyopathy, hypertrophic, 10 (OMIM 608758) **Spinal muscular atrophy with respiratory distress (OMIM 604320)**
83	Syria	1st cousins	One son homozygous for *GUCY2D* variant	1	Chr17(GRCh37):g.7917237G>A, NM_000180.3(GUCY2D):c.2303G>A, p.(Arg768Gln)	Leber congenital amaurosis 1 (OMIM 204000)
84	Afghanistan	1st cousins	Three healthy children, 2 deceased sons homozygous for *CEP290* variant	1	Chr12(GRCh37):g.88513990_88513994del, NM_025114.3(CEP290):c.1419_1423del, p.(Ile474Argfs*5)	Leber congenital amaurosis 10 (OMIM 611755)
85	Turkey	1st cousins	Two deceased children homozygous for *RMND1* variant, one living daughter homozygous for *RMND1* variant and one healthy son	2	Chr6(GRCh37):g.151738437G>C, NM_017909.3(RMND1):c.1177C>G, p.(Leu393Val) **Chr2(GRCh37):g.152471058A**>**G, NM_001164507.1(NEB):c.11333T**>**C, p.(Ile3778Thr)**	Combined oxidative phosphorylation deficiency 11 (OMIM 614922) **Nemaline myopathy 2, autosomal recessive (OMIM 256030)**
86	Afghanistan	1st cousins	Two deceased daughters homozygous for *ERCC6* variant	3	Chr10(GRCh37):g.50691430G>A, NM_000124.3(ERCC6):c.1954C>T, p.(Arg652*) **Chr3(GRCh37):g.48929490G**>**A, NM_000387.5(SLC25A20):c.121C**>T, p.(Gln41*) **Chr8(GRCh37):g.105441818C**>**T, NM_001385.2(DPYS):c.905G**>**A, p.(Arg302Gln)**	Cockayne syndrome, type B (OMIM 133540) **Carnitine-acylcarnitine translocase deficiency (OMIM 212138)** **Dihydropyrimidinuria (OMIM 222748)**
88	Turkey	1st cousins	One healthy daughter, one son homozygous for *HBB* variant	2	Chr11(GRCh37):g.5248178_5248184del, NM_000518.4(HBB):c.68_74del, p.(Glu23Valfs*37) **Chr19(GRCh37):g.45856060G**>**A, NM_000400.3(ERCC2):c.1846C**>**T, p.(Arg616Trp)**	β-thalassemia (OMIM 613985) **Xeroderma pigmentosum, group D (OMIM 278730)**
90	Netherlands	1st cousins		1	**Chr14(GRCh37):g.88452941T**>**C, NM_000153.3(GALC):c.334A**>**G, p.(Thr112Ala)**	**Krabbe disease (OMIM 245200)**
92	Iraq	1st cousins	One daughter homozygous for *CTNS* variant; 9 weeks pregnant at inclusion	1	Chr17(GRCh37):g.3563574G>A, NM_001031681.2(CTNS):c.1015G>A, p.(Gly339Arg)	Cystinosis, nephropathic (OMIM 219800)
93	Turkey	1st cousins	One healthy daughter, one deceased son homozygous for *ALPL* variant	1	Chr1(GRCh37):g.21889687G>A, NM_000478.5(ALPL):c.382G>A, p.(Val128Met)	Hypophosphatasia, infantile (OMIM 241500)
94	Morocco	1st cousins	Four sons, two healthy, two termination of pregnancy due to hydrops fetalis	1	**Chr17(GRCh37):g.18023748C**>**T, NM_016239.3(MYO15A):c.1634C**>**T, p.(Ala545Val)**	**Deafness, autosomal recessive 3 (OMIM 600316)**
95	Morocco	Distant	One son homozygous for *CHKB* variant	1	Chr22(GRCh37):g.51018188dup, NM_005198.4(CHKB):c.999dup, p.(Leu334Thrfs*95)	Muscular dystrophy, congenital, megaconial type (OMIM 602541)
96	Turkey	1st cousins	8 weeks pregnant at inclusion	1	**Chr11(GRCh37):g.17598421C**>**A, NM_001277269.1(OTOG):c.2604C**>A, p.(Cys868*)	**Deafness, autosomal recessive 18B (OMIM 614945)**
97	Afghanistan	1st/3rd cousins	One daughter homozygous for *TH* variant	1	Chr11(GRCh37):g.2185575G>A, NM_199292.2(TH):c.1475C>T, p.(Pro492Leu)	Segawa syndrome, recessive (OMIM 605407)
100	Iraq	1st cousins	One deceased daughter due to hydrocephalus, diagnosis unknown	1	Chr14(GRCh37):g.91739503dup, NM_001080414.3(CCDC88C):c.5553dup, p.(Ser1852Glnfs*4)	**Hydrocephalus, congenital, 1 (OMIM 236600)**

In bold: newly identified (novel) variants, in red: variants not (initially) identified by the current bioinformatics pipeline. ^ϯ^ indicates detection of compound heterozygous variants in the couple.

*AR* autosomal recessive, *ID* intellectual disability, *IUD* intrauterine death.

Finally, two novel findings in our series of 100 consisted of nonidentical variant carrier states potentially resulting in compound heterozygous variants in offspring, not associated with the consanguineous background but nevertheless a clinically significant finding of PCT (couples 63 and 66, Table 1).

Overall, 58/100 (58%) couples in our series are proven carrier couples for AR disease (Suppl. Table S1), of which 56 could be identified by our PCT (Table 1, Fig. 1a, Suppl. Fig. S1).

PCT initially confirmed 38 of the 45 previously known carrier states in 43 couples: 7 known carrier states were not (primarily) detected by PCT (Fig. 1a, Suppl. Table S4). There were four different reasons for this: (1) copy-number variants (CNVs) are not detected by ES (couple 23, wherein one parent carries two *SMN1* copies on one allele; couple 34, with a 3.4-kb deletion in the *HBA1* and *HBA2* genes; couple 63, with a 17p11.2 deletion), (2) delay in available literature not known at time of analysis (couples 7 and 62), (3) pipeline settings (couple 22 carries a deep intronic variant that is filtered out in the current ES analysis), and (4) exclusively registered with AD inheritance (couple 83).

Seven couples whose PCT results came back negative have offspring with a phenotype lacking a genetic diagnosis, such as rhabdomyolysis, congenital myopathy, or intellectual disability (Suppl. Table S1). Another three couples were shown to be carrier couples of diseases that did not explain their offspring’s phenotypes. The unidentified potential (genetic) causes of these phenotypes are diverse and do not necessarily derive from a failure of our analysis. At least some of the affected children underwent previous diagnostic ES that failed to identify any genetic cause.

### Follow-up (Fig. 1b, Fig. 2)

Couple 9 (*SUOX*) opted for PGT and are currently awaiting their first treatment. Couple 31 (*UPB1*) was pregnant at the moment the PCT result was available. PND showed the fetus to be unaffected by β-ureidopropionase deficiency. Previous offspring were tested and not affected.

**Fig. 2:**
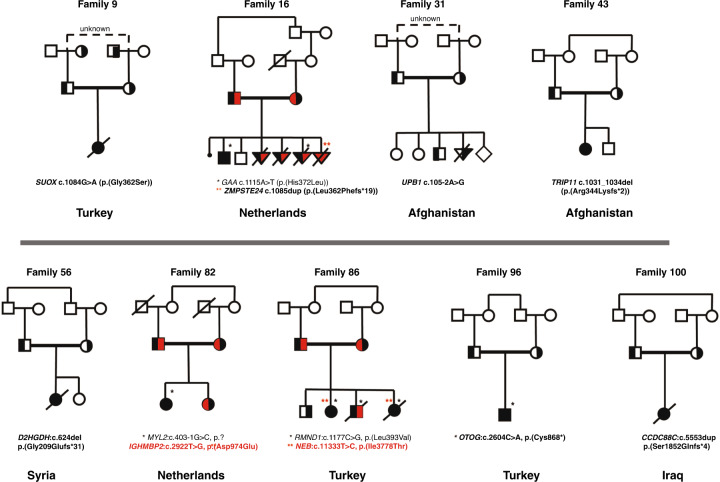
Family pedigrees described in more detail in the paper. For all pedigrees, see Supplementary Figure S1. *● homozygous for black variant,  homozygous red variant, half filled symbols are heterozygous variants.

For the six couples already enrolled in a PGT procedure where the PCT identified one or more additional disease carrier state, two (couples 4 and 22) decided to discontinue PGT, whereas four couples (2, 51, 82, 85) opted to add the additional disease risk to the PGT procedures. Couple 16 (*GAA* and *ZMPSTE24*) underwent PND for Pompe disease and lethal restrictive dermopathy. The fetus was affected by Pompe disease but not by restrictive dermopathy. The pregnancy ended spontaneously before a termination. In a subsequent pregnancy PND for both diseases showed that the fetus was affected by lethal dermopathy and the pregnancy was terminated. The couple is now opting for PGT for both disorders. Couple 82 (*MYL2* and *IGHMBP2*) tested their deceased and their healthy daughter for the *IGHMBP2* variant identified by PCT. Both were shown to be unaffected; the healthy daughter is heterozygous for the variant, the deceased daughter was not. Couple 85 (*RMND1* and *NEB*) had their children, who were affected by combined oxidative phosphorylation deficiency 11, tested for nemaline myopathy, which one deceased and one living child were shown to have (had) as well (homozygous for the variant). Their healthy son (*RMND1* heterozygote) does not carry the *NEB* variant. Couple 96 (*OTOG*) was pregnant when receiving the PCT result and did not opt for PND. The baby was tested postpartum and shown to be homozygous for the *OTOG* variant, and indeed deaf.

### Correlation between degree of consanguinity and number of shared variants

**Fig. 3:**
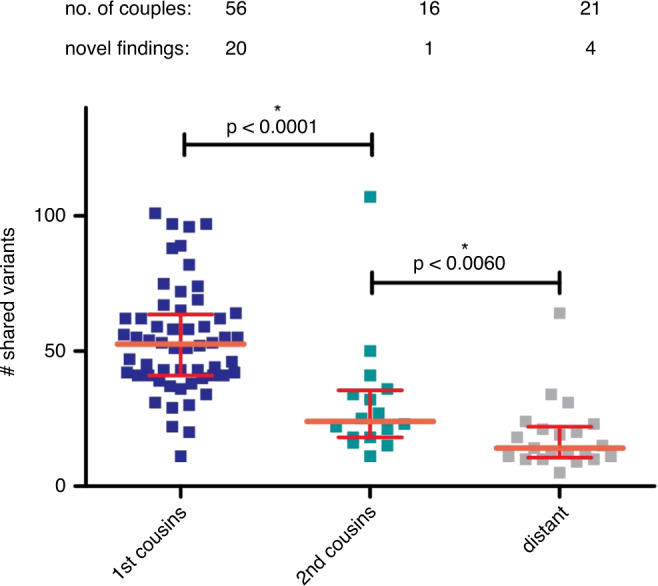
Correlation between degree of consanguinity and the observed number of identical variants shared between partners. Distant: all degrees of consanguinity farther removed than 2nd cousins. Novel findings: number of couples in which preconception carrier testing (PCT) detected at least one novel carriership. Crossbars: median with interquartile range. *Significant two-tailed *p* value (given) in Mann–Whitney *t*-test.

Unsurprisingly, we observed a correlation between an increasing degree of consanguinity and the number of identical variants shared between partners (Fig. 3). Of the 100 couples, 56 are first-degree relatives with an average of 54 shared identical variants. The 16 second cousin relationships shared on average 31 identical variants. Statistical testing (Mann–Whitney nonparametric) shows that the difference between these two groups is significant (*p* < 0.0001). The third grouping of 21 couples with a consanguineous relationship that is more distant than second cousins showed an average of 18 shared identical variants. This is statistically significant (*p* < 0.06) compared with the second cousin group. Novel variants were detected in 20 of the 56 first cousin couples (35.7%), in 1 of the 16 second cousin couples (6.3%), and in 4 of the 18 couples in the distant group (22.2%).

## DISCUSSION

We present the results of a diagnostic ES-based PCT in 100 consanguineous couples, showing that this test provides a powerful diagnostic tool for identification of AR disease carrier couple status. Outcomes provide significant reproductive choices for a higher proportion of consanguineous couples than other diagnostic tests currently offer.

Not counting the 45 previously known carrier states, our PCT results in an overall diagnostic yield for 30 novel carrier states in 28 couples (28%). The high diagnostic yield confirms the feasibility of a broad, ES-based approach in consanguineous couples. To the best of our knowledge, to date, no comprehensive studies have been published that provide insight into the proportion of consanguineous couples carrying recessive disease. Our study demonstrates that a considerable proportion of consanguineous couples in our cohort are carrier couples. One may argue that the couples who had a child with an undiagnosed phenotype that in retrospect was explained by the PCT result should not be taken into account in this figure in order to reduce bias. Excluding these four couples results in 24 newly identified carrier couples (24%) in our series. Still this is a significant number, which we found even in a heterogeneous group with varying degrees of consanguinity. Stratifying the data set stringently, limited to couples meeting a more formal definition of consanguinity, i.e., relatedness of second cousins or closer,^10^ would have resulted in a higher yield/risk figure, as can be seen in Fig. 3. Our data suggest that the empiric consanguinity-related health risk numbers used in genetic counseling may be an underestimation. However, our study is not a prospective study and bias can therefore not be excluded.

In total, 58/100 consanguineous couples in our series are carrier couples of at least one AR disease, 19 of which had already been included for PGT procedures.

As expected, the degree of consanguinity correlates with the absolute number of shared identical variants detected between partners in a couple. A more distantly related consanguinity results in significantly fewer shared variants but does not exclude a significant chance of identifying pathogenic carrier state.

The identification of two couples (2%) carrying a compound heterozygote disease risk demonstrates the potential for PCT application in the broader nonconsanguineous population and reflects a detectable disease risk based on population frequencies. Although compound heterozygous carrier states do not match the indication of consanguinity, they are obviously relevant to report, having the same relevance to the couples in terms of recurrence risk and reproductive choices as do homozygous carrier states.

In four couples PCT provided a genetic likely diagnosis for an affected, sometimes deceased, previous child, illustrating the potential usefulness of PCT in diagnosing deceased children without diagnosis (usually due to lack of available DNA of these deceased children).

Seven carrier states were not (initially) identified, mainly for technical reasons or due to unpublished literature at the time. Our PCT design builds on our previously implemented routine ES diagnostics,^19^ which because of technical limitations cannot guarantee 100% coverage of all exons of all genes and has a degree of mapping and alignment issues. Custom analysis depends on variant filter design that, for instance, limits analysis to positions +8 and −8 at exon–intron boundaries, excluding detection of deep intronic pathogenic variation. Performance limitations applying to ES in general may cause carrier state to elude the test. This is the reason a separate MLPA test for SMA is performed until validation of the *SMN1* exon 7–8 deletion detection in exome sequencing data can be completed (in progress). Such limitations are also the reason why, for example, *HBA1* and *HBA2* are excluded from our panel (couple 34), warranting separate ɑ-thalassemia testing in high-risk^20^ couples. Future developments will need to overcome these limitations.

Our panel design relies on well-defined genetic AR disease consensus as registered in OMIM and adequate and timely gene panel management. Any gene panel based approach requires continuous updating and curation, as is the case for our panel, but still has limitations. As illustrated in the series presented here, very recently discovered new causative disease genes that have not yet been registered in OMIM may be missed. The same applies to pathogenic variants that at the time of analysis are not yet reported in the literature or included in databases such as the Human Gene Mutation Database (HGMD).

The conceptual design of our test currently excludes CNV detection and AD and X-linked disease, although future directions in preconception health care may warrant inclusion.

Since we include virtually all known AR disease genes, the far higher diagnostic yield when compared with currently available PCS strategies (personal communication), working with small to medium-sized gene panels, is unsurprising.^21–23^ Our approach to include the highest possible number of disease-related genes was intentional, in order to maximize sensitivity in consanguineous couples who are at increased risk for any, including ultrarare, AR disorders.

In the context of PCS in general, focusing on severe diseases with childhood onset has been recommended in a statement of the European Society of Human Genetics (ESHG)^24^ and adopted by a national guideline, although the latter deliberately discourages categorical definitions of severe versus less severe. The American College of Obstetricians and Gynecologists (ACOG) proposes the following criteria for disease inclusion in a PCT: carrier frequency of >1 in 100, well-defined phenotype, detrimental effect on quality of life, cognitive or physical impairment, requiring surgical or medical intervention, early onset in life and exclude late onset disease, availability of intervention opportunities that result in improved outcomes, and education of parents about special care needs after birth.^25^ For a recently published selection of 1,300 genes for an Australian PCS project (Mackenzie’s Mission) the following inclusion criteria were applied: a condition should be life-limiting or disabling, with childhood onset, such that couples would be likely to take steps to avoid having an affected child; and/or be one for which early diagnosis and intervention would substantially change outcome.^26^ Of note, criteria may be (partly) different for general population screening panels compared with a PCT limited to consanguineous couples, which we present here. For example, for the very rare diseases that are more likely to be identified in consanguineous couples, a well-defined phenotype often is not available, as in many examples only one or a few cases have been described. This may, at least partially, explain why several genes in which carrier states were identified in our couples are not included in the Mackenzie’s list although they meet their abovementioned criteria (e.g., carnitine-acylcarnitine translocase deficiency [*SLC25A20*, OMIM 212138] and lethal congenital contracture syndrome [*NEK9*, OMIM 617022]): an additional selection criterion in the Mackenzie’s list is strong evidence that variants in the gene are associated with the condition in question.^26^ *MRPL44* (combined oxidative phosphorylation deficiency 16, OMIM 615395) and *SCAPER* (intellectual developmental disorder and retinitis pigmentosa, OMIM 618195) were not assessed by them and therefore not included, illustrating the difficulties in being as complete as possible despite an approach as thorough as theirs. Another potential challenge in testing consanguineous couples is that a pathogenic variant has been described in compound heterozygosity with another pathogenic variant, but never in a homozygous state. In such cases pathogenicity may be likely or evident but the phenotypic consequences less so. For our gene panel design, instead of making extensive choices based on interpretations of severity at the start, we opted to evaluate panel genes twice a year. In the first update we removed genes with unclear or very mild phenotypes, in later updates we mainly added novel AR genes described in literature (Suppl. Table S2).

The vast majority of carrier couple states we identified are indeed associated with serious disease, having impact on quality of life, causing impairment and/or requiring interventions, and with onset generally at infancy or early childhood (Table 1, Suppl. Table S3), thus meeting the ESHG criteria.

The classification of severity of disease has an inherent subjectivity^27^ and will remain controversial, particularly so in the context of PCT and reproductive (preventive) medicine. Local considerations rooted either in national law or cultural and social differences, are all codeterminants in the degree of severity definition, as is the actual availability of downstream preconception and/or prenatal diagnostic options in different parts of the world. Frequent gene panel updates based on expert consulting in our experience is an adequate tool for continuous re-evaluation and correction. For instance, the *HFE* gene (hemochromatosis type 1, OMIM 235200), initially included in the earliest version of the PCT gene panel, was reconsidered when actually encountered in an ongoing PCT analysis and, in a subsequent round of gene panel curation, was removed because of the adult onset and low penetrance characteristics of the associated pathology.

Other examples of the severity issue have been, e.g., congenital hearing loss or visual problems such as retinitis pigmentosa. Of note, AR congenital hearing loss caused by, for example, *GJB2* variants (DFN1B, OMIM 220290) is often categorized as moderately severe and not meeting several of the criteria discussed above.^28^ Still, in our center for PGT, it is one of the most frequently requested PGT indications (PGDnederland), adding patient experiences into the mix on the matter of disease severity opinion. One of the “mildest” disorders identified in our cohort was probably familial Mediterranean fever (FMF) (couple 66, Table 1, Suppl. Tables S1/S3). Although often with childhood onset, and potentially serious health complications, reproductive options will generally not be offered for FMF mainly due to its treatability. Ichthyosis vulgaris (couple 71, Table 1, Suppl. Tables S1/S3) is another debatable disorder in terms of severity, although the associated recessive disease has a more severe phenotype than dominant disease and PGT for other type congenital ichthyosis has been performed in our center, taking the treatment burden into consideration.

Once a couple is aware of their genetic risk(s), they can opt for reproductive choices such as refraining from having (further) children, accepting the risk, using donor gametes, or considering PND and PGT to avoid the birth of an affected child. Finally, couples may use the information to optimally prepare themselves for the birth of a potentially affected child, including choices for pre- and postnatal interventions to optimize outcome where applicable. The diverse options are illustrated by our follow-up data so far. Many couples testing positive for recessive disease carrier state in PCT will not have experience with the disease in their families, complicating their informed decision-making regarding available reproductive options.^29^ PCT-specific genetic counseling is essential and we have instigated several lines of clinical follow-up to aid in the development and improvement of these approaches. The lack of availability of an affected individual, currently needed for the development of a PGT laboratory protocol,^30^ requires development of novel PGT approaches. We and others are developing methods to directly phase the parental genomes, circumventing this requirement. Recently, PGT has already moved to genome-wide methods,^30–32^ allowing embryo analysis for multiple genetic defects with a single test instead of requiring multiple workups and analyses. This is clearly relevant in the context of consanguineous couples. Obviously, performing PGT for multiple disorders will yield a lower number of transferable embryos, potentially resulting in clinical and ethical dilemmas.^33^

Naturally, couples’ opinions about the PCT and the quality of PCT-related counseling and (after-) care is of eminent importance. We are currently conducting an extensive clinical follow-up study including in-depth interviewing techniques to gain more insight in this matter and aid in the adaptation of counseling practices.

### Conclusion

The results presented here show the clinical feasibility and utility of our ES-based comprehensive PCT approach for consanguineous couples. The high diagnostic yield emphasizes the benefit of including almost all AR disease genes, identifying the very rare carrier states consanguineous couples are particularly prone to. Recognizing their shared carrier status is of significant clinical importance for these couples, allowing them a well-informed reproductive choice. Our results open up avenues to future applications for this approach within the expert environment of clinical genetics. Extensive pretest counseling is essential.

## Supplementary information

Supplemental Legends

Supplementary Figure S1

Supplementary table S1

Supplementary table S2

Supplementary table S3

Supplementary table S4

## Data Availability

The data that support the findings of this study are available on request from the corresponding author (A.P.). The data are not publicly available due to privacy restrictions.

## References

[1] Bell CJ (2011). Carrier testing for severe childhood recessive diseases by next-generation sequencing. Sci. Transl. Med..

[2] Gulani, A. & Weiler, T. Genetics, autosomal recessive. (Treasure Island, FL, StatPearls, 2020). 31536227

[3] Fareed M, Afzal M (2017). Genetics of consanguinity and inbreeding in health and disease. Ann. Hum. Biol..

[4] Bennett RL (2002). Genetic counseling and screening of consanguineous couples and their offspring: recommendations of the National Society of Genetic Counselors. J. Genet. Couns..

[5] Kahrizi K (2019). Effect of inbreeding on intellectual disability revisited by trio sequencing. Clin. Genet..

[6] Oniya O, Neves K, Ahmed B, Konje JC (2019). A review of the reproductive consequences of consanguinity. Eur. J. Obstet. Gynecol. Reprod. Biol..

[7] Hamamy H (2012). Consanguineous marriages: Preconception consultation in primary health care settings. J. Community Genet..

[8] Hussain R (1999). Community perceptions of reasons for preference for consanguineous marriages in Pakistan. J. Biosoc. Sci..

[9] Shaw A (2014). Drivers of cousin marriage among British Pakistanis. Hum. Hered..

[10] Thain E (2019). Prenatal and preconception genetic counseling for consanguinity: consanguineous couples’ expectations, experiences, and perspectives. J. Genet. Couns..

[11] Modell B, Darr A (2002). Science and society: genetic counselling and customary consanguineous marriage. Nat. Rev. Genet..

[12] Bittles A (2001). Consanguinity and its relevance to clinical genetics. Clin. Genet..

[13] Sallevelt S, de Koning B, Szklarczyk R, Paulussen ADC, de Die-Smulders CEM, Smeets HJM (2017). A comprehensive strategy for exome-based preconception carrier screening. Genet. Med..

[14] Boycott KM (2017). International cooperation to enable the diagnosis of all rare genetic diseases. Am. J. Hum. Genet..

[15] Eaton A (2020). When to think outside the autozygome: best practices for exome sequencing in “consanguineous” families. Clin. Genet..

[16] de Ligt J (2012). Diagnostic exome sequencing in persons with severe intellectual disability. N. Engl. J. Med..

[17] Gilissen C (2014). Genome sequencing identifies major causes of severe intellectual disability. Nature.

[18] Richards S (2015). Standards and guidelines for the interpretation of sequence variants: a joint consensus recommendation of the American College of Medical Genetics and Genomics and the Association for Molecular Pathology. Genet. Med..

[19] Lelieveld SH (2016). Meta-analysis of 2,104 trios provides support for 10 new genes for intellectual disability. Nat. Neurosci..

[20] Piel FB, Weatherall DJ (2014). The alpha-thalassemias. N. Engl. J. Med..

[21] Beauchamp KA, Johansen Taber KA, Muzzey D (2019). Clinical impact and cost-effectiveness of a 176-condition expanded carrier screen. Genet. Med..

[22] Hoffman JD (2014). The Ashkenazi Jewish carrier screening panel: evolution, status quo, and disparities. Prenat. Diagn..

[23] Bristow SL (2019). Choosing an expanded carrier screening panel: comparing two panels at a single fertility centre. Reprod. Biomed. Online.

[24] Henneman L (2016). Responsible implementation of expanded carrier screening. Eur. J. Hum. Genet..

[25] Romero S, Rink B, Biggio JR, Saller DN (2017). Carrier screening in the age of genomic medicine. Obstet. Gynecol.

[26] Kirk EP (2020). Gene selection for the Australian Reproductive Genetic Carrier Screening Project (“Mackenzie’s Mission”). Eur. J. Hum. Genet..

[27] Lazarin GA, Haque IS (2016). Expanded carrier screening: a review of early implementation and literature. Semin Perinatol.

[28] Lazarin GA, Hawthorne F, Collins NS, Platt EA, Evans EA, Haque IS (2014). Systematic classification of disease severity for evaluation of expanded carrier screening panels. PLoS One.

[29] Vaz-de-Macedo C, Harper J (2017). A closer look at expanded carrier screening from a PGD perspective. Hum. Reprod..

[30] Drusedau M (2013). PGD for hereditary breast and ovarian cancer: the route to universal tests for BRCA1 and BRCA2 mutation carriers. Eur. J. Hum. Genet..

[31] Masset H (2019). Multi-centre evaluation of a comprehensive preimplantation genetic test through haplotyping-by-sequencing. Hum. Reprod..

[32] Zamani Esteki M (2015). Concurrent whole-genome haplotyping and copy-number profiling of single cells. Am. J. Hum. Genet..

[33] van der Schoot V (2019). Preimplantation genetic testing for more than one genetic condition: clinical and ethical considerations and dilemmas. Hum. Reprod..

