# Supplementary material for: Diagnostic exome-based preconception carrier testing in consanguineous couples: results from the first 100 couples in clinical practice
Source: Genet Med. 2021 Mar 19;23(6):1125–36. doi: 10.1038/s41436-021-01116-x (PMC8187149; doi:10.1038/s41436-021-01116-x)
Supplement: Supplementary file 1 — Supplemental Legends [file 41436_2021_1116_MOESM1_ESM.docx]

**Supplementary Figure S1**: Pedigrees of the couples with PCT findings (confirmed and newly identified carrier couple states).

**Supplementary Table S1**: Clinical and genetic data of all 100 consanguineous couples. *Known in famliy* relates to an affected child in the family proven to be homozygous for the described variant.

^ϯ^ indicates detection of compound heterozygous variants in the couple.

**Supplementary Table S2**: Gene panel version updates from DG2.13 to DG2.17

**Supplementary Table S3**: Disease categories of newly identified carrier couple states in our series

**Supplementary Table S4**: Known carrier couple states not detected with ES-PCT
