# Supplementary material for: Diagnostic exome-based preconception carrier testing in consanguineous couples: results from the first 100 couples in clinical practice
Source: Genet Med. 2021 Mar 19;23(6):1125–36. doi: 10.1038/s41436-021-01116-x (PMC8187149; doi:10.1038/s41436-021-01116-x)
Supplement: Supplementary file 2 — Supplementary Figure S1 [file 41436_2021_1116_MOESM2_ESM.pdf]

Supplementary Figure S1

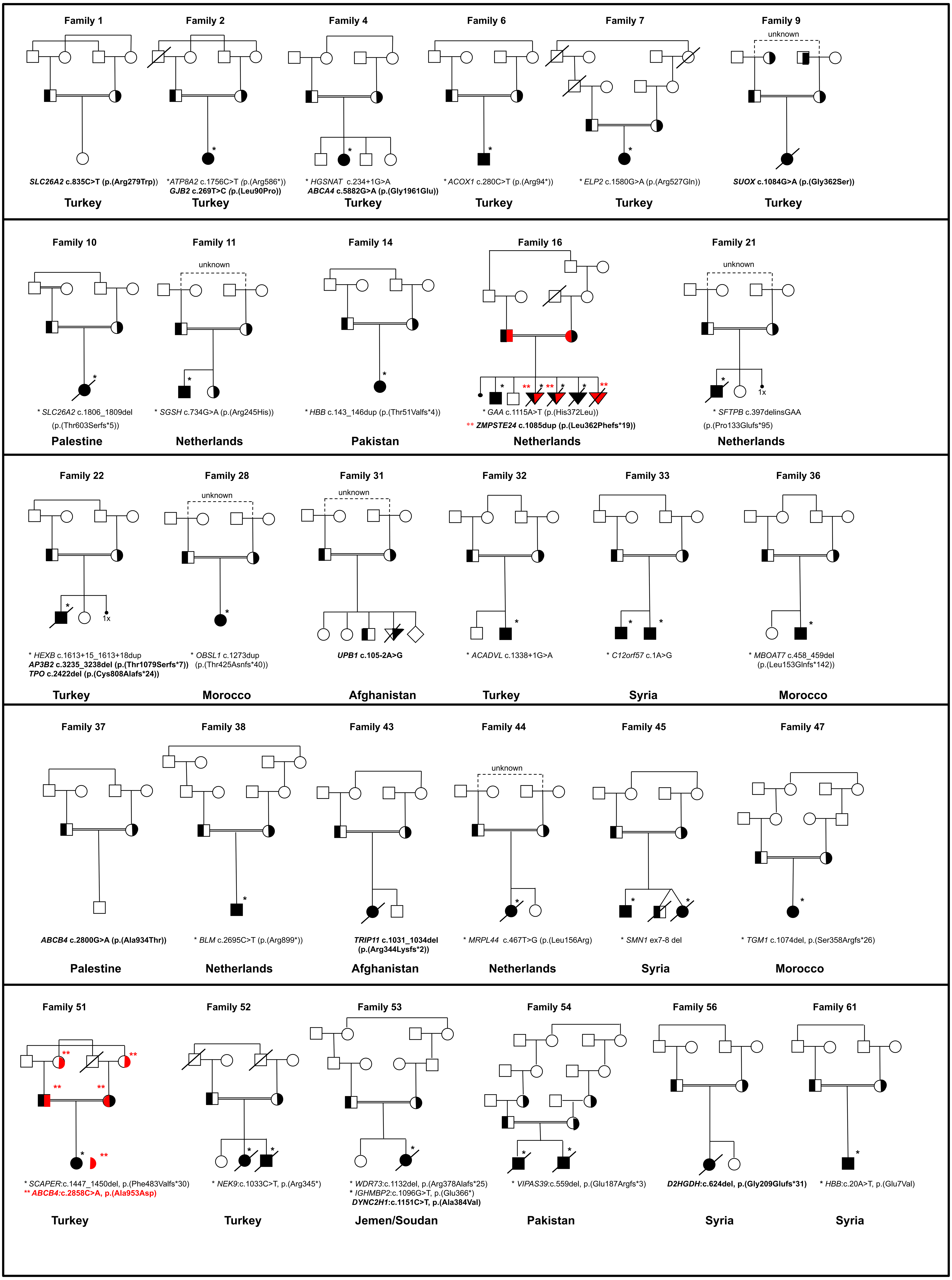

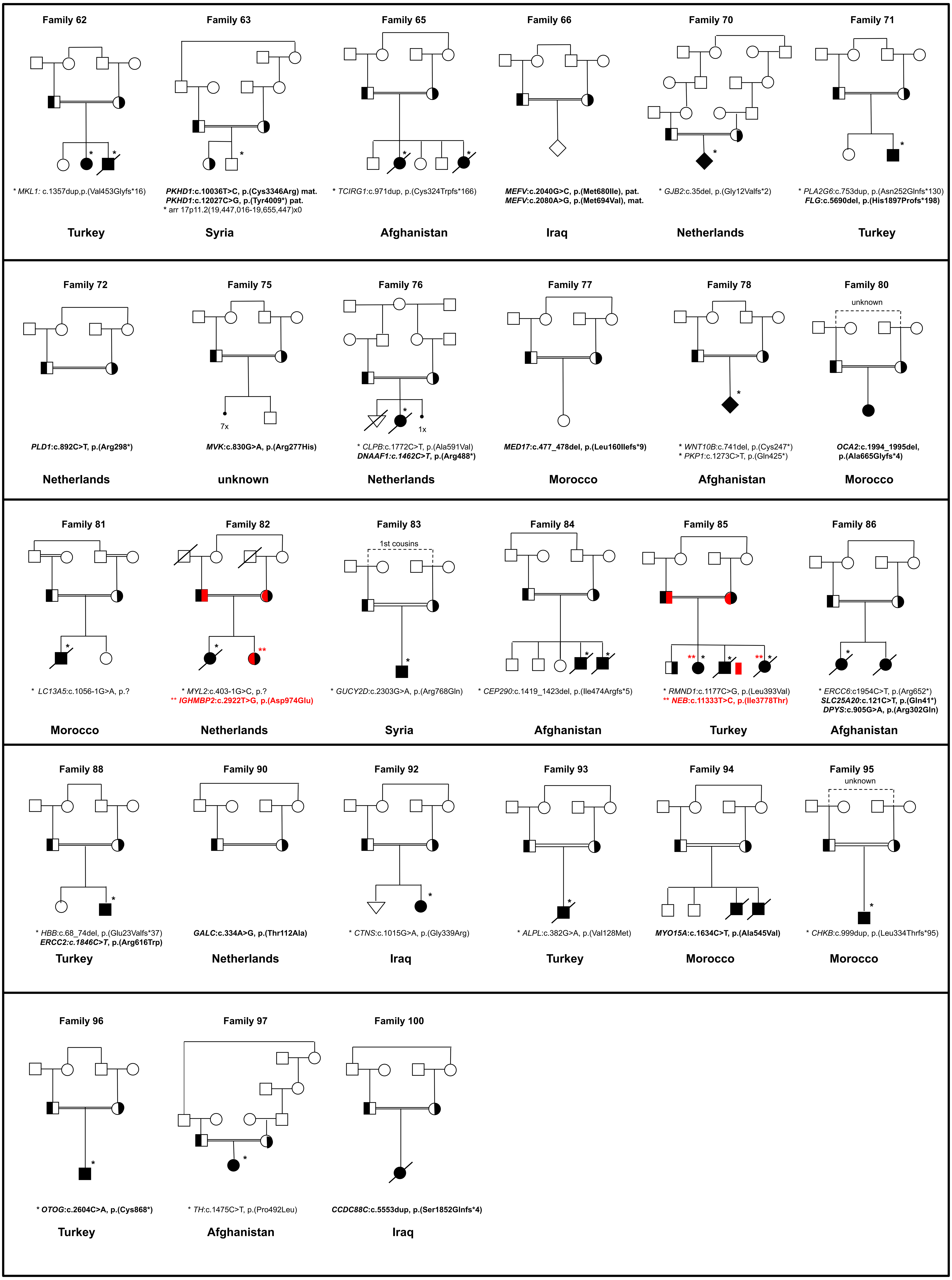

Family 62
* MKL1: c.1357dup,p.(Val453Glyfs*16)
Turkey
Family 63
PKHD1:c.10036T>C, p.(Cys3346Arg) mat.
PKHD1:c.12027C>G, p.(Tyr4009*) pat.
* arr 17p11.2(19,447,016-19,655,447)x0
Syria
Family 65
* TCIRG1:c.971dup, p.(Cys324Trpfs*166)
Afghanistan
Family 66
MEFV:c.2040G>C, p.(Met680Ile), pat.
MEFV:c.2080A>G, p.(Met694Val), mat.
Iraq
Family 70
* GJB2:c.35del, p.(Gly12Valfs*2)
Netherlands
Family 71
* PLA2G6:c.753dup, p.(Asn252Glnfs*130)
FLG:c.5690del, p.(His1897Profs*198)
Turkey
Family 72
PLD1:c.892C>T, p.(Arg298*)
Netherlands
Family 75
7x
MVK:c.830G>A, p.(Arg277His)
unknown
Family 76
1x
* CLPB:c.1772C>T, p.(Ala591Val)
DNAAF1:c.1462C>T, p.(Arg488*)
Netherlands
Family 77
MED17:c.477_478del, p.(Leu160Ilefs*9)
Morocco
Family 78
* WNT10B:c.741del, p.(Cys247*)
* PKP1:c.1273C>T, p.(Gln425*)
Afghanistan
Family 80
unknown
OCA2:c.1994_1995del,
p.(Ala665Glyfs*4)
Morocco
Family 81
* LC13A5:c.1056-1G>A, p.?
Morocco
Family 82
* MYL2:c.403-1G>C, p.?
** IGHMBP2:c.2922T>G, p.(Asp974Glu)
Netherlands
Family 83
1st cousins
* GUCY2D:c.2303G>A, p.(Arg768Gln)
Syria
Family 84
* CEP290:c.1419_1423del, p.(Ile474Argfs*5)
Afghanistan
Family 85
* RMND1:c.1177C>G, p.(Leu393Val)
** NEB:c.11333T>C, p.(Ile3778Thr)
Turkey
Family 86
* ERCC6:c1954C>T, p.(Arg652*)
SLC25A20:c.121C>T, p.(Gln41*)
DPYS:c.905G>A, p.(Arg302Gln)
Afghanistan
Family 88
* HBB:c.68_74del, p.(Glu23Valfs*37)
ERCC2:c.1846C>T, p.(Arg616Trp)
Turkey
Family 90
GALC:c.334A>G, p.(Thr112Ala)
Netherlands
Family 92
* CTNS:c.1015G>A, p.(Gly339Arg)
Iraq
Family 93
* ALPL:c.382G>A, p.(Val128Met)
Turkey
Family 94
MYO15A:c.1634C>T, p.(Ala545Val)
Morocco
Family 95
unknown
* CHKB:c.999dup, p.(Leu334Thrfs*95)
Morocco
Family 96
* OTOG:c.2604C>A, p.(Cys868*)
Turkey
Family 97
* TH:c.1475C>T, p.(Pro492Leu)
Afghanistan
Family 100
CCDC88C:c.5553dup, p.(Ser1852Glnfs*4)
Iraq
